# RSM-based Model to Predict Optimum Fermentation Conditions for Soluble Expression of the Antibody Fragment Derived from 4D5MOC-B Humanized Mab in SHuffle™ T7 *E. coli*

**DOI:** 10.22037/ijpr.2020.114377.14822

**Published:** 2021

**Authors:** Aidin Behravan, Atieh Hashemi

**Affiliations:** *Department of Pharmaceutical Biotechnology, School of Pharmacy, Shahid Beheshti University of Medical Sciences, Tehran, Iran.*

**Keywords:** Box-Behnken design, Response surface methodology, 4D5MOC-B scFv, Culture conditions optimization, EpCAM, SHuffle™ T7

## Abstract

Overexpression of the EpCAM in epithelial-derived neoplasms makes this receptor a promising target in antibody-based therapy. Due to the lack of N-glycosylation, *Escherichia coli* (*E. coli*) seems to be the most appropriate choice for the expression of antibody fragments. However, developing a robust and cost-effective process that produces consistent therapeutic proteins from inclusion bodies is a major challenge. Undoubtedly, it can be circumvented by the soluble expression of these proteins. Utilization of numerous genetically modified hosts and optimization of cultivation conditions are two effective approaches widely used to overcome the insolubility problem. Due to the cytoplasmic expression of *DsbC* and the ability to the correct formation of disulfide bonds, the Shuffle™ T7 strain can be a suitable host for the soluble production of recombinant proteins. Here, Box-Behnken design (BBD)- Response surface methodology (RSM) modeling was employed to develop optimized culture conditions for 4D5MOC-B scFv fragment production in SHuffle™ T7 strain while solubility and production level were considered as responses. Although both responses were significantly influenced by post-induction temperature, cell density at induction time, and IPTG concentration, the temperature had the largest effect. The maximum experimental soluble protein obtained by adding 1 mM of IPTG into the M9 medium when the cell density reached 0.7 at 23 ᵒC was 693.56 µg/mL which was in good correlation with the predicted value of 720.742 µg/mL. Predictable total expression value was also experimentally verified. This strategy can be scaled-up for the production of large amounts of scFvs from SHuffle™ T7 *E. coli* to facilitate their potential applications as therapeutic and diagnostic agents.

## Introduction

Among monoclonal antibody fragments, a single-chain variable fragment (scFv) is a well-studied molecule. ScFv is an artificial fusion protein consisting of the variable domains of heavy (V_H_) and light (V_L_) chains of an antibody joined by an artificial flexible polypeptide of 10–25 amino acids (1). Due to their small molecular weights, they can penetrate tumor tissues better than their parental antibodies. Owing to their small size along with ease of genetic manipulation, they can be a suitable alternative to intact antibodies for a variety of diagnostic and therapeutic purposes.

Although varying expression hosts were employed for scFv production, *Escherichia coli* (*E. coli*) is the most frequently used system for the production of these unglycosylated small molecules. However, high-level expression in the cytoplasm of *E. coli* usually contributes to intracellular protein aggregation and inclusion body formation which necessitate additional refolding steps. One of the major challenges during refolding the protein from a denatured state is the inactive protein formation (2). Optimization of cultivation conditions is one of the main strategies widely used to circumvent the insolubility problem (3). The utilization of numerous genetically modified hosts is another effective approach that can be considered. For example, to facilitate correct protein folding within the cytoplasm, the SHuffle™ T7 *E. coli* strain was established in which disulfide bond formation can be catalyzed due to thioredoxin reductase (*trxB*) and glutathione reductase (*gor*) mutations. Moreover, cytoplasmic expression of *DsbC*, a chaperone disulfide bond isomerase, in this strain has allowed disulfide bonds in proteins to be reshaped inside the cytoplasm. Consequently, proper formation of disulfide bonds leads to lesser accumulation of misfolded proteins and inactive inclusion body formation (4).

As mentioned earlier, the high level of soluble protein expression can also be achieved by careful optimization of culture conditions such as temperature, isopropylβ-D-1-thiogalactopyranoside (IPTG) concentration, and cell density at induction time (3). In culture conditions optimization based on the one-factor-at-a-time method, many trial-and-error experiments are required until an optimum is reached. However, the true optimal conditions for the best response may not be achieved. Moreover, this strategy is time and labor-consuming and interactions between the different variables can’t be identified (5). Identifying significant factors and interactions between them are two major advantages of DoE circumventing limitations of the OFAT method during optimization. This statistical approach can result in a more reliable prediction of the true optimum via a reduced number of experiments (6). Many studies used response surface methodology (RSM) of DoE to successfully achieve high yields in recombinant protein production (7, 8). RSM can model the effects of individual variables and their interactions. Using this method, multivariable equations can be solved and the importance of several input factors towards the desired outcome can be evaluated. For model optimization, Box-Behnken design (BBD) and Central Composite Design (CCD) are two generally used designs (9). In many studies, both upstream and downstream processes of protein production have been optimized based on RSM-Box-Behnken design (6, 10 and 11).

In the current study, the culture conditions have been applied based on RSM-Box-Behnken design for improving both total expression and solubility of 4D5MOC-B scFv fragment in the SHuffle™ T7 *E. coli* strain. Three physiochemical parameters; cell density at induction time, induction temperature, and IPTG concentration and their effects, alone and in combination, were examined. 4D5MOC-B scFv is a high affinity and very stable anti-EpCAM extracellular domain-scFv (antiEpEX-scFv) generated from the binding residues of parental hybridoma MOC31 which was grafted onto the scFv 4D5 framework (3). EpCAM is one of the first target antigen for tumor immunotherapy because of its overexpression in epithelial-derived neoplasms (12). We previously showed higher solubility of 4D5MOC-B scFv when expressed in SHuffle™ T7 *E. coli* compared to BL21^TM^ (DE3) (13). So, for the first time, Box-Behnken design was applied to obtain optimal setting of the variables which can influence total protein expression or solubility of 4D5MOC-B scFv in SHuffle™ T7 *E. coli* in this study. Moreover, M9, a chemically defined minimal medium was used for antiEpEX-scFv production in shake flask which is more interesting for industrial-scale production fermentations than a complex medium like LB because feeding strategy is controllable and culture conditions are reproducible when it is used. Besides, this minimal medium is less expensive than a complex one. So this would make the 4D5MOC-B scFv production more economically viable for an industrial scale-up (14).

## Experimental

*Bacterial strains and growth media*

*E. coli* strain (DH5α) (gifted by Dr. Keramati, Pasteur institute of Iran, Tehran, Iran) harboring the pET22b-antiEpEX-scFv vector (3) was used as a cloning host. *E. coli* strain (SHuffle™ T7) (gifted by Dr. Nematollahi, Pasteur institute of IRAN, Tehran, Iran) was employed as the bacterial host for the expression of the recombinant scFv.

One-hundred milliliter M9 chemically defined minimal media containing 0.337 mM Na_2_HPO_4_, 0.22 mM KH_2_PO_4_, 0.08 mM NaCl, 0.093 mM NH_4_Cl, 0.01 mM CaCl_2_, 0.2 mM MgSO_4_, 0.01 mL 1000x trace element (Teknova), was complemented with 4 g/L D-glucose, 0.05 mM thiamine hydrochloride (Sigma-Aldrich) and ampicillin (100 μg/mL). One liter Luria-Bertani (LB) medium including 10 g/L tryptone, 5 g/L yeast extract, and 10 g/L NaCl was also used.

*Expression of the 4D5MOC-B scFv*

To investigate if 4D5MOC-B scFv can be expressed in M9 minimal media in *E. coli* strain (SHuffle™ T7), the expression plasmid pET22b (+)-4D5MOC-B scFv was transformed into chemically competent *E. coli* SHuffle™ T7 cells with heat shock method and a single colony of SHuffle™ T7 *E. coli *harboring pET22b (+)-4D5MOC-B scFv was cultured overnight at 37 °C in LB broth supplemented with 100 µg/mL ampicillin as a pre-culture. Then 50 mL of M9 minimal medium was inoculated with 10% (v⁄v) of the pre-culture and then incubated at 30 °C until reaching OD_600_ = 0.6 which was followed by adding 1 mmol/L isopropyl-b-D-thiogalactopyranoside (IPTG) as inducer, After 24 h of induction, cells were harvested by centrifugation (10000 *×g*; 10 min at 4 °C) while refrigeration was used to keep it at −20 °C for next analysis. Culture condition optimization experiments were performed based on RSM-BBD experimental design. After validation of protein expression in M9 minimal medium, different experiments based on RSM-BBD were performed to optimize culture conditions.

*Protein isolation and analysis*

Cells were harvested by centrifugation of culture broth at 10000 g for 10 min at 4°C, followed by resuspension in 20 mL of lysis buffer (1 mg/mL lysozyme, 20 mM Tris pH 7.5, 50% glycerol, 50 mM NaCl). Then after 40 min incubation on ice, the cells were disrupted by sonication for 30 min (20 s on/5 s off) at 400 W and centrifuged at 15,000 *×g* for 30 min at 4 °C. Then the supernatant was collected as a soluble fraction and the pellet was collected as an insoluble fraction. SDS-PAGE was used to illustrate and analyze the expression level of the recombinant scFv. At the end of the process, the gel was stained by coomassie brilliant blue G-250. ImageJ software (NIH, MD) was used to quantify the expressed protein by image analysis method.

*Purification of the soluble portion of recombinant 4D5MOC-B scFv fragment*

Bacterial cells were harvested and suspended in lysis buffer (50 mM NaCl, 20mM Tris HCl pH 7.5, 1 mg/mL lysozyme, 50% glycerol). The container was vortexed and sonicated (400 W for 18 min 20 s ON, 10 s OFF). The soluble fraction was separated from insoluble debris by centrifugation at 10000 *×g* for 25 min at 4 °C. The supernatant fraction was suspended in a denatured buffer (Tris 50 mM, NaCl 50 mM, 1% triton X100, 8 M Urea; pH 8) and subjected to the Ni–NTA agarose affinity chromatography column under denatured condition according to manufacturer instruction (Qiagen, Netherlands). Using buffers containing 20 mM imidazole, the Ni–NTA column was washed and then the antiEpEX-scFv was eluted from the column by 250 mM imidazole. The purified protein concentration was determined by a bicinchoninic acid (BCA) protein assay kit (Takara, Japan).

*4D5MOC-B scFv quantification*

Calculation of the 4D5MOC-B scFv concentration was accomplished using the linear regression equation of Bovine Serum Albumin (BSA) protein as a standard. To draw the standard curve, discrete values of 0; 0.01; 0.05; 0.125; 0.250; and 0.500 mg/mL were used as concentration levels while their relative intensities had been measured using ImageJ software from their SDS-PAGE results. The linear regression equation is then calculated based on the obtained curve. To quantify the 4D5MOC-B scFv expression level in all experiments, high-quality pictures were captured from the SDS-PAGE gels and analyzed by the ImageJ software, then the recombinant 4D5MOC-B scFv concentration was calculated using the linear regression equation y = ax + b (which had been extracted from the standard curve). Where the intensity of the protein was the independent variable (x) and the concentration was the dependent variable (y) (15).

*Experimental design and statistical analysis*

Using the factorial RSM-BBD methodology, the effects of three independent factors; post-induction temperature, inducer concentration, and cell density at induction time, on the final total protein expression and solubility of 4D5MOC-B scFv fragment, were systematically examined in this study (Table 1). A three-factor-three-level BBD consisted of 15 experiments is presented in Table 2. Design of the experiments and statistical analysis of the results were accomplished using the software package Design_Expert version 11^®^ (Stat-Ease Inc., Minneapolis, USA). Moreover, to evaluate the validity of the model presented in results, the combination of tested variables leading to the maximum predicted level of the final total protein expression and soluble fraction of 4D5MOC-B scFv fragment was experimentally validated (16).

## Results

*Expression of 4D5MOC-B scFv fragment*

**Figure 1:**
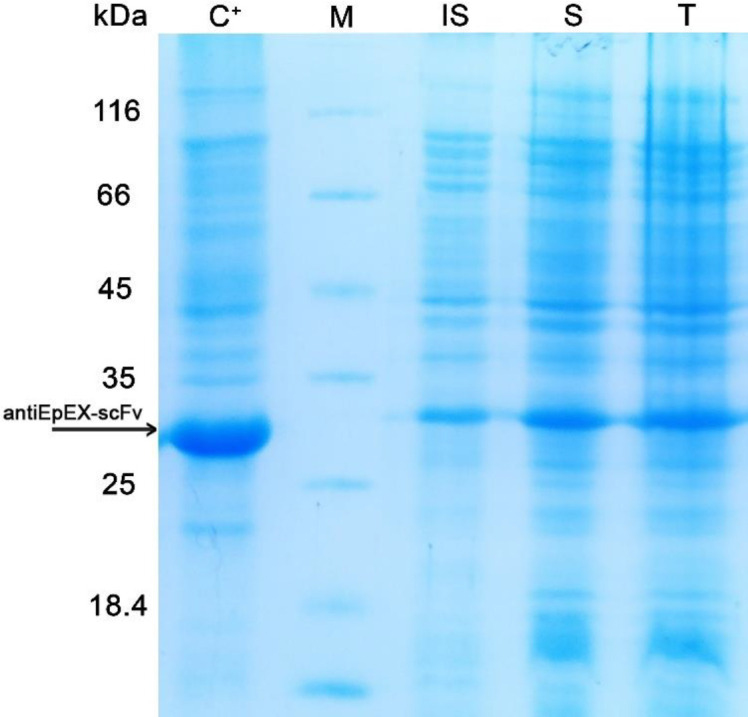
SDS-PAGE analysis of antiEpEX-scFv expression in *E. coli* SHuffle™ (DE3) in M9 minimal medium. (IS) Insoluble Fraction, (S) Soluble fraction, (T) Total lysate of antiEpEX-scFv protein expression induced inOD_600_ = 0.6 with 1 mM IPTG at 30 ᵒC for 24 h in M9 minimal medium. (C^+^): induced total cell lysate of *E. coli* SHuffle™ (DE3) in OD_600_ = 0.8 with 0.8 mM IPTG at 37 ᵒC for 24 h in LB medium, (M) Protein molecular weight marker (14.4 – 116 kDa). Arrow indicated antiEpEX-scFv (~ 30 kDa).

Before optimization, to examine the level of the 4D5MOC-B scFv fragment expression in *E. coli* strain SHuffle™ T7, 5 mL of the pre-cultured cells harboring pET22b (+)-4D5MOC-B scFv was inoculated into 50 mL of M9 minimal medium and induced at mid-log phase (OD_600_ of 0.6 nm) with 1mM IPTG at 30 ᵒC for 24 h. Following induction with IPTG, the bacterial cells were lysed by ultrasonication. The cell lysate before centrifugation, centrifugal supernatant, and pellet fractions were analyzed by SDS-PAGE. The recombinant protein was detected in both soluble and insoluble fractions (Figure 1) and existed in soluble form as 88–90% of the total 4D5MOC-B scFv fragment.

*RSM based optimization of the 4D5MOC-B scFv fragment expression; on the total protein expression and solubility*

RSM-BBD methodology was successfully utilized to investigate the individual and interaction effects of post-induction temperature, IPTG concentration, and cell density at induction time on the total 4D5MOC-B scFv fragment expression and solubility in M9 minimal medium. The three-level BBD with 15 runs was employed (Table 2). Table 2 summarizes the results achieved from the BBD experiments for the total 4D5MOC-B scFv fragment expression and solubility. Based on the experimental data, quadratic models represented by the following equations can describe the correlation between total 4D5MOC-B scFv fragment expression (μg/mL) (Equation 1) or solubility (Equation 2) and three various variables.

y=antiEpEX-scFv µgml= 3.70554e+06 + 763544 A -280951 B + 843876 C -1.50655e + 06 AB + 404552 AC -442516 BC -410083 A^2^ + 133683 B^2^ + 264911 C^2^

Equation 1.

(R^2^ = 0.9887, R^2^adj = 0.9684)

y=antiEpEX-scFv µgml= 759.446 + 105.509 A -48.4557 B + 195.645 C -285.748 AB + 31.7792 AC -62.6628 BC -30.478 A^2^ + 56.9127 B^2^ + 24.9006 C^2^

(R^2^ = 0.9838, R^2^adj = 0.9545)

Equation 2.

Where Y is the response (antiEpEX-scFv expression level), and A, B and C are letters used for IPTG concentration, OD_600_nm before induction, and the temperature respectively. Based on designed experiments, a wide range of results (350.07 to 1092.55 μg/mL) was obtained for the soluble expression level and (454.667 to 1229.030 μg/mL) for the total expression level of the recombinant protein for all 15 experiments (Table 2). The highest total protein level (1229.030 μg/mL) was obtained when cells were induced at 30 ᵒC with 0.1 mM IPTG for 24 h.

*Statistical analysis of the model*

**Figure 2:**
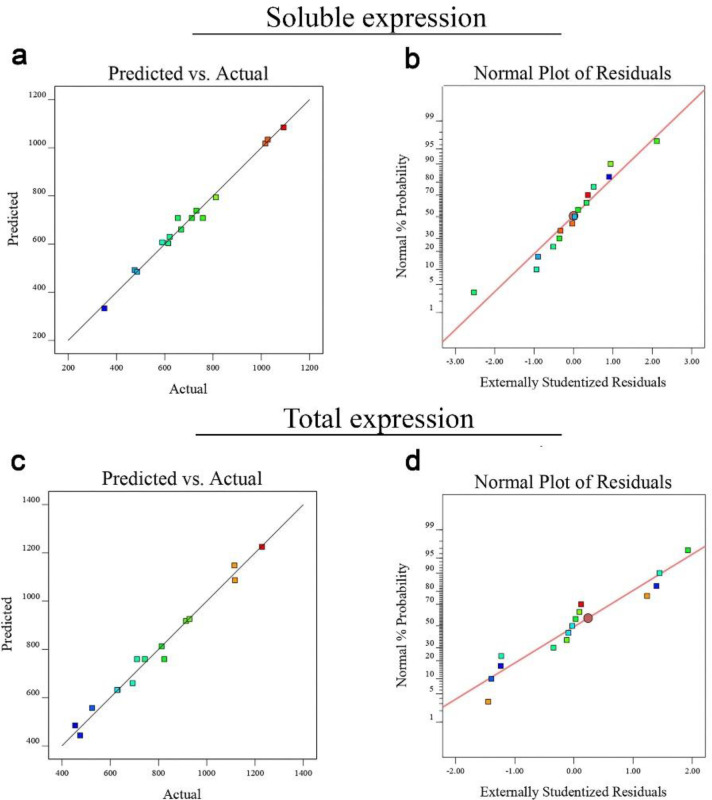
The diagnostic plots for the quadratic models. (a) Predicted *vs*. actual plots; (b) normal plot (soluble expression); (c) Predicted *vs.* actual plots; (d) normal plot (total expression).

Using the Design-Expert software, quadratic regression models developed for optimization of the total 4D5MOC-B scFv fragment expression as well as solubility were confirmed by analysis of variance (ANOVA). The results of ANOVA values for solubility and protein expression (μg/mL) are represented in Supplementary Tables 1 and 2 respectively. The F-value of the model developed for optimization of the 4D5MOC-B scFv fragment solubility (48.73) implies that the model is significant. There is only a 0.02% chance that an F-value of the proposed model can occur due to the noise. For total 4D5MOC-B scFv fragment expression, ANOVA also showed the significance level of the model (F-value = 33.66). The Low *P*-value (0.0006) of the model resignifies the significance of the model. Moreover, the mathematical analysis demonstrated that the lack of fit values (*P* = 0.8855, and 0.6703 for solubility and total protein expression responses respectively) of the models were not significant, indicating the suitable predictive performance of selected models. The R^2^ values for the models developed for optimization of the antiEpEX-scFv solubility and total protein expression were found to be 0.9887 and 0.9838 respectively, which show that values predicted by RSM models are very well correlated with the experimental data. Furthermore, the values of the Predicted R² are in reasonable agreement with the values of Adjusted R² in both developed models. As shown in normal probability plots of the studentized residuals, the significance and adequacy of the proposed models were also confirmed by the linear trend of data points (Figure 2). Moreover, predicted and experimental values are in good agreement for both responses studied here (Figures 2a and 2c). As a result, these models can be used to navigate the design space.

*Influence of parameters on the total protein production of the recombinant 4D5MOC-B scFv fragment*

**Figure 3:**
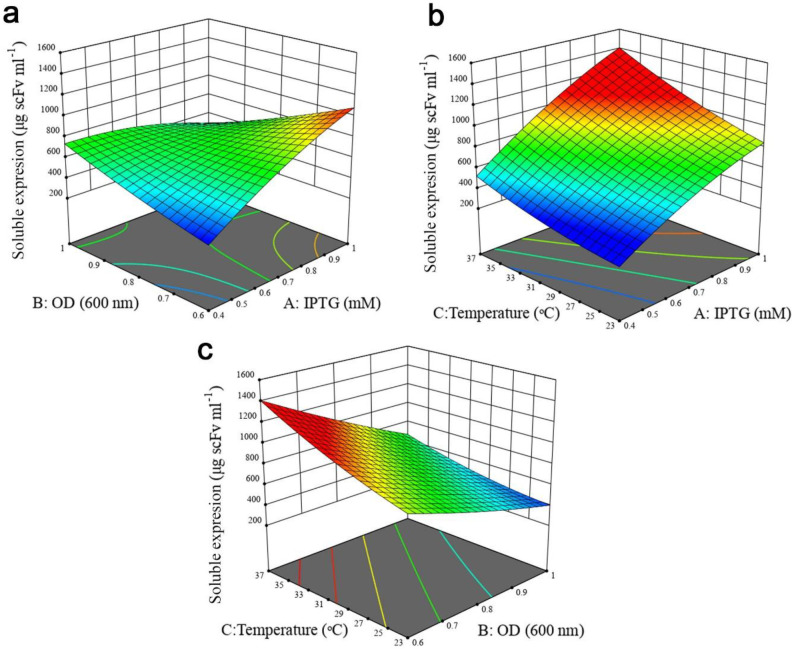
Response surface plots to illustrate the interaction of experimental variables on the antiEpEX-scFv soluble expression level using the BBD-RSM methodology. (a) OD_600_ and IPTG concentration; (b) post induction temperature and IPTG concentration; (c) OD_600_ and post induction temperature. Units in response plots are μg scFv mL^−1^

**Figure 4:**
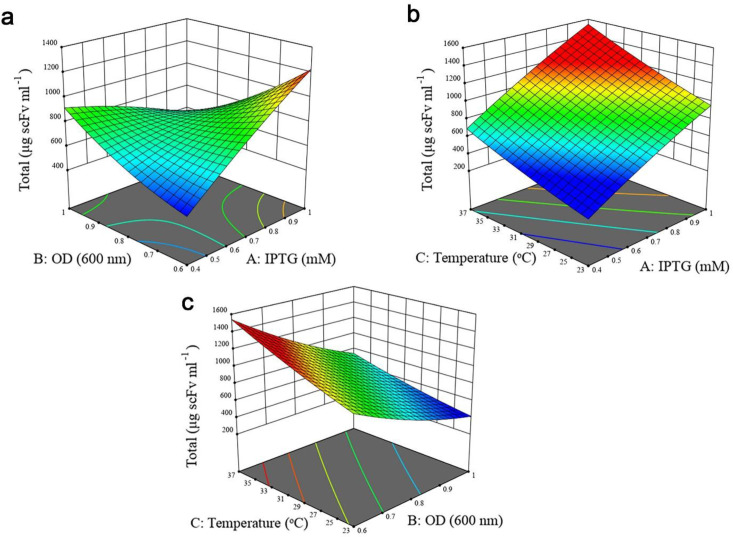
Response surface plots to represent the interaction of experimental variables on the antiEpEX-scFv total expression level using the BBD-RSM methodology. (a) OD_600_ and IPTG concentration; (b) post induction temperature and IPTG concentration; (c) OD_600_ and post induction temperature. Units in response plots are μg scFv mL^−1^.

A *P*-value less than 0.05 was considered statistically significant. For solubility response, except B2 and C2, all the linear (A, B, and C), quadratic (A^2^) and interactive terms (AB, AC, and BC) were significant as observed from the *P*-values of the model terms. In the case of total protein expression, all the linear terms of the model and the interactive effect of IPTG concentration- cell density before induction were found to be significant, whereas quadratic (A^2^, B^2,^ and C^2^) and the interactive effect of cell density before induction- post-induction temperature, and IPTG concentration- post-induction temperature indicated no significant impact. Furthermore, it was concluded that post-induction temperature had the largest effect on both responses, followed by IPTG concentration and OD_600_nm before induction. The interactive effects between independent variables on two responses were studied using three-dimensional response surface graphs. Based on ANOVA results (Tables S1 and S2, Supplementary file), the interaction of IPTG concentration and OD_600_nm before induction was significant (*P* < 0.0001). As illustrated in Figure 3a, when induction temperature was kept at 30 °C, protein solubility increased with both variables. Although increasing cell density before induction at lower IPTG concentration enhanced antiEpEX-scFv solubility, an increase in OD_600_ at higher IPTG concentration had a negative effect on the soluble expression of the protein. According to the low *P*-value (0.0161) of the coefficient AC (Table S1, Supplementary file), there is also a significant interaction between IPTG concentration and induction temperature. As depicted in Figure 3b, antiEpEX-scFv soluble expression increased with IPTG concentration, while the increase in induction temperature had only a minor effect on protein solubility. Although increasing the temperature has a low effect on the soluble expression of the protein at lower IPTG concentration, it will be more effective at a higher concentration of the inducer. As depicted by the low *P*-value of the coefficient BC of 0.0114 (Table S1, Supplementary file), there is significant interaction between these two variables. The dependency of the temperature and OD_600_nm before induction on protein solubility when the concentration of IPTG was kept constant (0.7) is shown in Figure 3c. According to this graph, more solubility was obtained by the increase in temperature, while protein soluble expression decreased with the increase in OD_600_nm. Figure 4a illustrates the positive effect of IPTG concentration and OD_600_nm before induction on the total expression of recombinant protein while the temperature was kept at 30 °C. However, an increase in OD_600_nm at higher IPTG concentration led to a decrease in total protein production. Figure 4b declares the positive impact of concentration of IPTG and temperature when OD_600_nm was kept constant (0.7). Total protein expression significantly was enhanced with IPTG concentration, especially when the temperature was high. Interestingly, based on ANOVA results, the interaction of these variables was insignificant due to the high *P*-value of the coefficient AC (*P* = 0.2654). Finally, the high *P*-value of the coefficient BC of 0.0564 indicates that the interaction between OD_600_ and temperature is not significant (Table 3). Figure 4c explains that the total protein production of the antiEpEX-scFv protein will be improved by increasing temperature while the increase in the value of the OD_600_ has a negative effect on the total protein expression.

*Validation of the total protein expression and solubility models*

**Figure 5:**
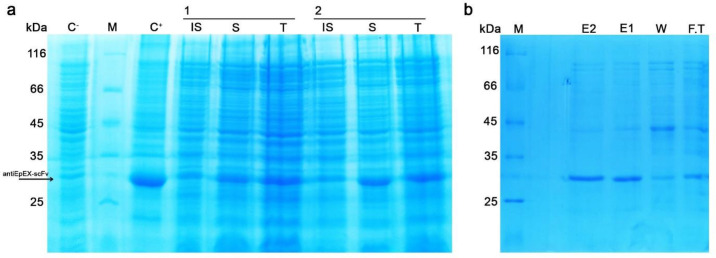
SDS-PAGE analysis to characterize the optimum conditions. (a) two repeats of antiEpEX-scFv expression for model confirmation (induced with 1 mM IPTG in OD_600_ = 0.7 at 23 ᵒC), (C^+^): induced total cell lysate of *E. coli* SHuffle (DE3) in LB medium, (C^-^) uninduced total cell lysate, (IS): insoluble fraction, (S): soluble fraction, (T): Total cell lysate, (M) Protein molecular weight marker (14.4 – 116 kDa); (b) purified antiEpEX-scFv with Ni-NTA column, (E1, E2) Eluted proteins fractions, (W) wash , (F.T) flow through (M) Protein molecular weight marker (14.4 – 116 kDa).

The optimal culture conditions as achieved from the solubility model at the maximum point were calculated to be as 1, and 0.7 for IPTG concentration, cell density before induction (OD_600_), and post-induction temperature of 23 ᵒC, respectively. The maximum predicted value of 720.74 μg/mL was in good correlation with the experimentally obtained value 693.56 μg/mL predictable total expression value was also experimentally verified. Using the predicted optimum conditions determined by the solubility model, experiments were also conducted in duplicates for validation of the total expression model. Validation of the total expression model contributes to the total 4D5MOC-B scFv fragment expression of 842.51 μg/mL, which is close to the predicted production of 781.952 μg/mL (Figure 5a). So, the accuracy of the BBD models was fully confirmed. Moreover, using the Ni-NTA affinity chromatography column, the recombinant antiEpEX-scFv protein was purified from soluble fraction followed by BCA analysis. 281.578 μg/mL of the soluble protein was obtained in optimum fermentation conditions (Figure 5b).

## Discussion

Although *E. coli* is still the preferred choice system for recombinant protein production among all available hosts, the expression of proteins with several disulfide bonds in this cell factory is challenging. Due to the reducing environment of the cytoplasm, most the expressed heterologous proteins tend to form aggregates and insoluble inclusion bodies (4, 17). Despite high yield production, easy separation from other cellular components, and resistance to proteases, costly optimization is needed to make them be solubilized and re-folded *in-vitro*. Hence, efforts have been made to devise practical strategies for the effective and inexpensive production of soluble proteins (18). Circumventing the insolubility problem, utilization of engineered hosts such as SHuffle™ T7 strain with the more oxidizing environment can contribute to disulfide bond formation and proper folding (19). Employing this approach, in our study high-level accumulation of recombinant protein was detected in the soluble fraction as 86–88% of the total protein. In line with our results, recombinant EhCP1 enzyme was expressed in SHuffle™ T7 strain is fully soluble and active form (20). Moreover, a high amount (2600 mg/L) of soluble DsbA-IGF1 was obtained in the cytoplasm of this strain (6).

As parameters including IPTG concentration, temperature and cell density at induction time have been effective on production level and solubility of recombinant proteins, here, RSM-BBD methodology was utilized to optimize the expression level and solubility of 4D5MOC-B scFv fragment. Based on previously published data, Box-Behnken design was reported to be more efficient than the central composite design as well as full-factorial designs (21). The high F-value, as well as low *P*-value of the models developed in the current study for optimization of the protein solubility (F = 48.73, *P *= 0.0002) and total expression (F = 33.66, *P *= 0.0006) confirmed that these mathematical models were significant. The suitable predictive performance of selected models was also supported by their insignificant lack of fit values. Consistently, Akbari et al showed that BBD methodology could efficiently be applied for optimal production of anti-Her2-scFv. In their study, the highest yield of recombinant protein was obtained when cells were induced at 37 ᵒC with 0.75 mM IPTG for 10.45 h (16). Anti-keratin scFv TS1-218 was optimally produced by Jafari and coworkers also (21-fold increase) in culture conditions formulated via RSM-BBD methodology. They evaluated the impact of temperature, methanol concentration, and pH on protein expression (22). Moreover, the successful optimization of DsbA-IGF1 expression and purification was reported by Emamipour *et al.* utilizing BBD-RSM methodology (6).

Although all studied parameters were significant for production level and soluble expression of 4D5MOC-B scFv fragment in the current study, the post-induction temperature had the largest effect on both responses, with higher temperatures giving rise to increased protein expression level, but lower antiEpEX-scFv solubility. Higher solubility at low temperatures was previously reported for several recombinant proteins including Fab fragments, human interferon α-2 ricin A chain, subtilisin E, and β-lactamase (18, 23). In the current study, the maximum level of soluble 4D5MOC-B scFv fragment was obtained at 23 °C. Similar results were previously observed with DsbA-IGF1 protein. Emamipour *et al.* applied the BBD methodology to achieve a high concentration of correctly folded protein. According to their results, the maximum solubility was obtained at 23 °C resulting in 2600 mg/L fusion protein (6). When lowering the expression temperature, the rates of cell processes such as transcription, translation, and cell division will be reduced allowing enough time to form correctly folded proteins. Moreover, at a lower temperature, the expression and activity of many chaperones are increased facilitating proper folding of the proteins (3). On the other hand, as was shown here, a higher temperature (33 °C) was more proper for obtaining the most recombinant protein expression level. In line with our results, in Ahmadzadeh *et al.* study, anti-HER2 scFv was maximally expressed at 30 °C in SHuffle™ T7 strain (4). Besides, Volontθ and colleagues reported that the recombinant protein was optimally expressed at a growth temperature of 37 °C (24). However, most of the protein was expressed as an inclusion body due to the temperature dependency of hydrophobic interactions.

A wide range of IPTG concentrations has been suggested for gene expression induction (0.005 to 5 mM) (25). High IPTG concentration can cause a decrease in growth rate and an increase in bacterial proteases leading to heterologous proteins degradation. Due to the potential toxicity of IPTG and high cost, its optimum concentration needs to be optimized (27). On the other hand, a low IPTG concentration commonly leads to low protein yield (16). Our results showed a significant effect of IPTG concentration on both total 4D5MOC-B scFv fragment expression and its solubility in SHuffle™ T7 strain. Here, the best conditions for both responses were achieved in 1 mM IPTG while we previously reported that antiEpEX-scFv fragment was optimally produced in *E. coli* BW25113 (DE3) and BL21 (DE3) strains in the presence of 0.8 mM and 0.5 mM of IPTG respectively (3). Similar results were obtained by Ahmadzadeh and coworkers. They reported that higher amounts of anti-HER2 scFv could be achieved in *E. coli* BL21 (DE3) induced with 0.25 mM IPTG while varying IPTG concentration had no significant effect on the production of this protein in SHuffle™ T7 *E. coli* (4). Consequently, the expression of one specific recombinant protein can be differently affected by IPTG concentration when heterologously expressed in various *E. coli* hosts.

**Table 1:** Independent variables and values of levels used in Box–Behnken experimental design

**Symbols**	**Variables**	**Units**	**Coded levels**		
			**-1**	**0**	**+1**
A	IPTG	(mM)	0.4	0.7	1
B	OD	(600 nm)	0.6	0.8	1
C	Temperature	(ᵒC)	23	30	37

**Table 2:** Factor values and response based on Box–Behnken experimental design

**Experiments**	**IPTG (A) (mmol/L)**	**OD (B) (600 nm)**	**Temperature (C) (ᵒC)**	**Soluble antiEpEX-scFv production (µg/mL)**		**Total antiEpEX-scFv production (µg/mL)**	
				**actual**	**predicted**	**actual**	**predicted**
1	0.4	0.8	23	485.41	484.80	454.667	484.49
2	0.7	0.6	23	589.71	607.62	628.792	631.41
3	0.7	1	23	668.02	661.16	693.080	659.82
4	1	0.8	23	614.21	603.77	631.136	631.95
5	0.7	0.8	30	654.40	708.17	743.696	759.45
6	0.4	1	30	731.47	738.94	914.231	917.66
7	0.4	0.6	30	350.07	332.77	475.521	443.08
8	1	1	30	475.41	492.70	524.743	557.19
9	0.7	0.8	30	758.00	708.17	823.657	759.45
10	1	0.6	30	1092.55	1085.08	1229.030	1225.59
11	0.7	0.8	30	712.12	708.17	710.984	759.45
12	0.4	0.8	37	619.96	630.40	813.042	812.22
13	0.7	1	37	812.08	794.17	928.400	925.79
14	1	0.8	37	1016.89	1017.50	1116.630	1086.80
15	0.7	0.6	37	1027.07	1033.93	1114.760	1148.02

**Table 3:** Confirmation of optimization results

**Condition **	**IPTG (mmol/L)**	**OD (600 nm)**	**Temperature (°C)**	**Soluble antiEpEX-scFv production (µg/mL)**		**Total antiEpEX-scFv production (µg/mL)**	
				**actual **	**predicted **	**actual **	**predicted **
Validation	1	0.7	23	693.56	720.742	842.51	781.952

## Conclusion

In summary, firstly, a valuable 4D5MOC-B scFv fragment was efficiently produced in SHuffle™ T7 *E. coli* in a chemically defined minimal medium. For large-scale production of recombinant proteins, M9 is the preferred medium because it can provide process reproducibility and the final product quality. However, most of the published data is based on the cultivation of rich media. Secondly, the expression level and solubility of protein were optimized via selecting appropriate variables and their levels. Using the statistical design of experiments, the maximum soluble protein was obtained 693.56 μg/mL which was in good correlation with the predicted value 720.74 μg/mL. Predictable total expression value was also experimentally verified. The optimized culture conditions obtained here could be applied to bioreactor-based high cell density fermentation for soluble production of 4D5MOC-B scFv fragment.

## Supplementary Materials

Supplement
