# Supplementary material for: RSM-based Model to Predict Optimum Fermentation Conditions for Soluble Expression of the Antibody Fragment Derived from 4D5MOC-B Humanized Mab in SHuffle™ T7 E. coli
Source: Iran J Pharm Res. 2021 Winter;20(1):254–66. doi: 10.22037/ijpr.2020.114377.14822 (PMC8170757; doi:10.22037/ijpr.2020.114377.14822)
Supplement: Supplement [file ijpr-20-254-s001.pdf]

Supplementary Materials for

RSM-based Model to Predict Optimum Fermentation Conditions for Soluble Expression of the Antibody Fragment Derived from 4D5MOC-B Humanized Mab in SHuffle™ T7 *E. coli*

Aidin Behravan and Atieh Hashemi*

*To whom correspondence should be addressed. E-mail: at_hashemi@sbmu.ac.ir

Volume 20, Issue 1 (Winter 2021)

This PDF file includes:

Tables S1 and S2

Figure S1

Table S1. ANOVA quadratic model for soluble fraction.

Source	Sum of Squares	df	Mean Square	F-value	*P*-value	
Model	2.253E+13	9	2.504E+12	48.73	0.0002	significant
A-IPTG	4.664E+12	1	4.664E+12	90.78	0.0002	
B-OD	6.315E+11	1	6.315E+11	12.29	0.0172	
C-Temperature	5.697E+12	1	5.697E+12	110.88	0.0001	
AB	9.079E+12	1	9.079E+12	176.70	< 0.0001	
AC	6.546E+11	1	6.546E+11	12.74	0.0161	
BC	7.833E+11	1	7.833E+11	15.24	0.0114	
A^2	6.209E+11	1	6.209E+11	12.09	0.0177	
B^2	6.599E+10	1	6.599E+10	1.28	0.3085	
C^2	2.591E+11	1	2.591E+11	5.04	0.0747	
Residual	2.569E+11	5	5.138E+10			
Lack of Fit	6.059E+10	3	2.020E+10	0.2058	0.8855	not significant
Pure Error	1.963E+11	2	9.815E+10			
Cor Total	2.279E+13	14				

Table S2. ANOVA quadratic model for Total Expression.

Source	Sum of Squares	df	Mean Square	F-value	*P*-value	
Model	7.789E+05	9	86539.90	33.66	0.0006	significant
A-IPTG	89057.03	1	89057.03	34.64	0.0020	
B-OD	18783.62	1	18783.62	7.31	0.0426	
C-Temperature	3.062E+05	1	3.062E+05	119.11	0.0001	
AB	3.266E+05	1	3.266E+05	127.04	< 0.0001	
AC	4039.67	1	4039.67	1.57	0.2654	
BC	15706.51	1	15706.51	6.11	0.0564	
A^2	3429.81	1	3429.81	1.33	0.3003	
B^2	11959.57	1	11959.57	4.65	0.0835	
C^2	2289.39	1	2289.39	0.8905	0.3887	
Residual	12854.66	5	2570.93			
Lack of Fit	6135.03	3	2045.01	0.6087	0.6703	not significant
Pure Error	6719.63	2	3359.82			
Cor Total	7.917E+05	14				

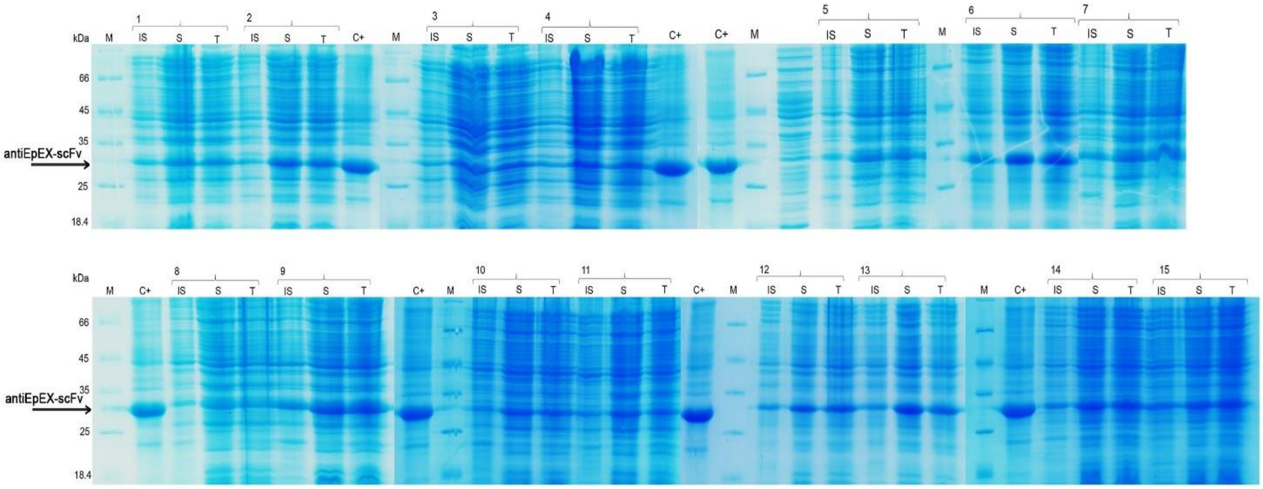

Figure S1. SDS-PAGE analysis of antiEpEX-scFv expression in different experiments according to Box–Behnken Design. M: Protein molecular weight markers, 1–15: experimental conditions, (IS: insoluble fraction; S: soluble fraction; T: Total cell lysate). (C^+): induced total lysate of *E. coli* SHuffle (DE3) in LB medium. (arrow): recombinant antiEpEX-scFv protein [~ 30 kDa].
